# Genome-Wide ChIP-seq and RNA-seq Analyses of STAT3 Target Genes in TLRs Activated Human Peripheral Blood B Cells

**DOI:** 10.3389/fimmu.2022.821457

**Published:** 2022-03-08

**Authors:** Jing Wu, Ying-Ying Jin, Ruo-Lan Gong, Fan Yang, Xiao-Ya Su, Tong-Xin Chen

**Affiliations:** ^1^ Division of Immunology, Pediatric Translational Medicine Institute, Shanghai Children’s Medical Center, School of Medicine, Shanghai Jiao Tong University, Shanghai, China; ^2^ Allergy/Immunology Innovation Team, Shanghai Children’s Medical Center, School of Medicine, Shanghai Jiao Tong University, Shanghai, China; ^3^ Department of Rheumatology/Immunology, Shanghai Children’s Medical Center, School of Medicine, Shanghai Jiao Tong University, Shanghai, China

**Keywords:** ChIP sequencing, RNA sequencing, Toll-like receptors, B cells, STAT3

## Abstract

Toll like receptors (TLRs) induced response plays a vital role in B-cell development and activation, in which TLR7-mediated and TLR9-mediated response interact together and play antagonistic or cooperative roles at different situations. Previous studies showed that the transcription factor signal transducer and activator of transcription (STAT) 3 was one of the key transcriptional factors (TFs) needed for both TLR7 and TLR9 signaling in B cell, and patients with autosomal dominant hyper IgE syndromes (AD-HIES) due to *STAT3* mutations having defective TLRs response in B cells. However, how STAT3 affects its target genes and the downstream signaling pathways in B cell upon TLRs stimulation remains unclarified on a genome-wide level. ChIP-seq and RNA-seq was used in this study to identify the STAT3 targets in response to TLRs stimulation in human B cell. STAT3 ChIP-seq results showed a total of 611 and 2,289 differential STAT3-binding sites in human B cell after TLR7 and TLR9 agonists stimulation, respectively. RNA-seq results showed 1,186 and 1,775 differentially expressed genes after TLR7 and TLR9 activation, respectively. We identified 47 primary STAT3 target genes after TLR7 activation and 189 target genes after TLR9 activation in B cell by integration of STAT3 ChIP-seq and RNA-seq data. Among these STAT3 primary targets, we identified 7 TFs and 18 TFs for TLR7 and TLR9 response, respectively. Besides, we showed that STAT3 might regulate TLR9, but not TLR7 response in B cells through directly regulating integrin signaling pathway, which might further affect the antagonism between TLR7 and TLR9 signaling in B cell. Our study provides insights into the molecular mechanism of human TLRs response in B cell and how it can be regulated, which helps to better understand and modulate TLR-mediated pathogenic immune responses in B cell.

## Introduction

B cell plays a central role in adaptive immunity, whose activation is tightly controlled by four kinds of receptors that include B cell receptors (BCRs), cytokine receptors, receptors involved in cognate T cell–B cell interactions, and innate immune receptors, namely, Toll-like receptors (TLRs) (1–3). In the past decades, accumulating pieces of evidence has demonstrated the importance of TLRs in the B-cell development and activation, and their role in the pathogenesis of autoimmune disease and B-cell malignancies (1–4). Human B cell mainly express the endosomal TLR7 and TLR9 that are involved in the recognition of RNA and DNA from microbial-derived nucleic acid or endogenous nucleic acid released from damaged or dying cells, respectively (3, 5). Under normal physiological conditions, B cell recognizes and makes a rapid immune response against invading foreign microbes. Ligation of TLR7 and TLR9 promotes the B-cell proliferation, activation, immunoglobulin and cytokines secretion, and its maturation into memory B cell (6). However, aberrant activation of B cell incited by excessive endogenous nucleic acid released by damaged or dying cells could lead to the generation of autoantibodies, and subsequent autoimmune diseases. Besides, antagonism between TLR7 and TLR9 in B cell was well demonstrated in both *in vivo* and *in vitro* studies, in which TLR9 plays protective roles against autoimmunity through inhibiting TLR7-mediated autoantibody production (2). Therefore, aberrant TLRs activation in B cell was not only reported in patients with primary immunodeficient diseases, such as hyper-IgE syndrome (HIES) (6, 7) and common variable immunodeficiency disease (CVID) (5, 8), but also autoimmune diseases, namely, systemic lupus erythematosus (SLE) and Sjögren’s syndrome (9, 10).

Hyper IgE syndromes (HIES) comprise a group of rare primary immunodeficiency disorders, which are characterized by extremely high serum IgE levels, eczema, recurrent skin, and pulmonary infections (11). Autosomal dominant HIES (AD-HIES), which is the most common form of this disease, is caused by *STAT3* gene mutations (12). Defective TLR9-driven B cell proliferation and immunoglobulin production has been reported in AD-HIES patients in the previous studies, implicating the vital role of STAT3 in the TLR9-stimulated B-cell response (6, 7). We recently reported that, TLR9-driven upregulation of co-stimulatory molecules CD40, CD80, and CD86, and the production of Interleukin 10 (IL10), was also defective in B cell from AD-HIES patients (13). More interesting, not only TLR9-, but also TLR7-driven B-cell response, was observed to be defective in AD-HIES patients (13), suggesting that STAT3 played an important role not only in TLR9-, but also TLR7-induced B cell response. Taken together, these studies suggested that STAT3 plays a vital role in TLR7/9-mediated immune response in B cell, and TLR7/9 share STAT3 to mediate, at least part of these immune response.

To date, however, how STAT3 affects its target genes and the downstream signaling pathways in B cell upon TLRs stimulation remains unclarified. Figuring out the STAT3-regulated genes and its downstream signaling is important to finding novel therapy to rescue or compensate the defective TLRs response in B cell from patients with AD-HIES. Besides, identification of downstream targets of STAT3 in human B cell in response to TLRs activation is of particular interest, because STAT3 is not only involved in TLRs-mediated, but also BCR- and cytokine-mediated activation of B cell (2, 7, 14). Moreover, further exploration of functional differences between TLR7 and TLR9 during B cell activation, and the potential mechanism underlying their pathways in B cell will help to development of agents, which could potentially prevent infection and autoimmune states in patients.

Herein, in this study, we used a combination of genome-wide analysis methods and computational data integration, which enabled us to identify direct and indirect STAT3 targets in response to TLRs activation in B cell. It will help us to have a better understanding of the functional differences between TLR7 and TLR9 during B-cell activation, and the vital role of STAT3 involved in these immune responses.

## Materials and Methods

### Human CD19^+^ B Cell Preparation and Purification

CD19^+^ B cells were purified from peripheral blood mononuclear cell (PBMC) from healthy donors with EasySep™ Human B Cell Isolation Kit (StemCell technologies) according to the instructions of the manufacturer. Briefly, PBMC were isolated from peripheral blood by gradient density centrifugation. CD19^+^ B cells were then isolated by negative selection by using CD19 beads. The purity of CD19 B-cell used was over 95% in all experiments. The study was approved by the local ethical institute (Shanghai Children’s Medical Center, School of Medicine, Shanghai Jiao Tong University).

### B Cell Treatment With TLR Agonists

To examine the TLR induced response in B cell, purified CD19^+^ B cells were stimulated with TLR7 agonist (R848, 2.5 ug/ml, InvivoGen) or TLR9 agonist (CpG ODN 2006, CpG for short, 5 μg/ml, InvivoGen) in 96-well round bottom plates in RPMI 1640 medium (Gibco) supplemented with 10% Fetal bovine serum (FBS, Gibco) and 1% penicillin–streptomycin (Hyclone) for 24 h.

### STAT3 ChIP-seq Studies

ChIP assay was carried out as previous studies (15, 16). Briefly, after crosslinking chromatin with 1% formaldehyde for 10 min and neutralizing with 0.125 M glycine for 5 min at room temperature, cells were washed with cold PBS, and lysed in cold lysis buffer on ice. The cell lysate was centrifuged, and the precipitation was suspended with 0.8 ml of radioimmunoprecipitation assay (RIPA) buffer. After that, the cell lysate was sonicated, and then incubated with anti-STAT3 antibody precoated on Dynabeads protein G (Life Technologies) at 4°C overnight. Immunoprecipitated products were collected, washed with RIPA buffer twice, and LiCl washing buffer for three times. Then, the immunoprecipitated products were digested with Proteinase K, and the formaldehyde crosslinks were reversed. The purified DNA fragments were sent for high-throughput sequencing.

Reads from ChIP-seq were aligned to the hg19 reference genome using Bowtie software (17). The numbers of STAT3 raw peaks are 14,645, 10,483, and 15,974 in the unstimulated B cell, R848 treated B cells and CpG treated B cells, respectively (p-value = 1e−5). After filtering ambiguously-mapped and duplicates reads, STAT3-binding sites in TLRs-activated B cell relative to the unstimulated B cell (control) were identified using the MACS tool with default parameter settings (18). Bigwig files were generated using bamCoverage from the deeptools package (version 3.5.0) with parameters “-e 150 -bs 10 –normalizeUsing RPKM”. Results from the ChIP-seq were visualized in Integrative Genomics Viewer (19).

### RNA-seq and Quantitative RT-PCR

CD19^+^ B cells were treated with TLRs agonist for 24 h, then the cells were collected, washed with PBS twice, and lysed with TRIzol reagent for the following RNA-seq in BGI Biotechnology. Total RNA was qualified using an Agilent 2100 Bioanalyzer, and DNBSEQ platforms were used to perform a high-throughput sequencing. RNA-seq transcript data was analyzed using the TopHat/Cufflinks combination (20).

To detect the expression of Interleukin 7 (*IL-7*) after TLRs activation in B cell, purified B cells were treated with R848 or CpG for 24 h. The primer sequence of human *IL-7* is as follows: forward: GCGAGCAGCACGGAATAAAA, reverse: AGTGTTCTAATGGTCAGCATC G.

### B-Cell Proliferation

B-cell proliferation was detected by CFSE cell proliferation assay according to the previous study (13). Briefly, PBMC were incubated in 5 μmol/l CFSE at 37°C for 10 min, and washed with PBS supplemented with 5% FBS. The CFES-labeled cells were treated with R848 or CpG for 5 days. Then the cells were harvested, washed, and stained with CD19-APC-H7 (BD) according to the instructions of the manufacturer. Samples were then analyzed using BD FACS Canto II (BD, USA).

### Luciferase Reporter Assay

IL-7 promoter sequence was amplified by PCR from human genomic DNA, and the primer sequence of IL-7 is as follows: forward: GAAAGCTAGCAGGGTCCTGGGAGTGACTAT; reverse: GAAACTCGAGACGTTGGCTATTCTTTCCGC. The PCR product was purified and cloned in to the pGL3-promoter vector.

Luciferase reporter assay was performed according to the previous study (15). Briefly, 100 ng pGL3-IL-7 promotor plasmid was co-transfected with 20 ng of pRL-SV40 renilla luciferase reporter, different concentrations of pcDNA3.1-STAT3 plasmid (0, 50, 100, 200, and 400 ng), and different concentrations of empty vector of pcDNA3.1 (400, 350, 300, 200, and 0 ng) using FuGENE^®^ 6 Transfection Reagent (Promega). Forty-eight hours after transfection, luciferase activity was determined by using a commercial dual luciferase reporter assay system (Promega) according to the instructions of the manufacturer.

### Enzyme Linked Immunosorbent Assay (ELISA)

*IL-7* production induced by TLRs agonist in B cell was quantified using ELISA. Briefly, 1 × 10^5^ purified B cell in PBMC from 3 healthy donors was seeded in round-bottom 96-well plate, R848 or CpG was used to treated cells for 5 days, then the concentrations of *IL-7* in supernatants were quantified using human *IL-7* ELISA kit (Dakewe Biotech) according to the instructions of the manufacturer.

### Statistics

Values are expressed as mean values ± standard deviation. Data were analyzed with Prism 6.0 (GraphPad Software) using one-way ANOVA or unpaired t-test. *P <*0.05 was considered statistically significant.

## Results

### STAT3 is Required for TLRs Induced Response in Human Peripheral Blood B Cell

**Figure 1:**
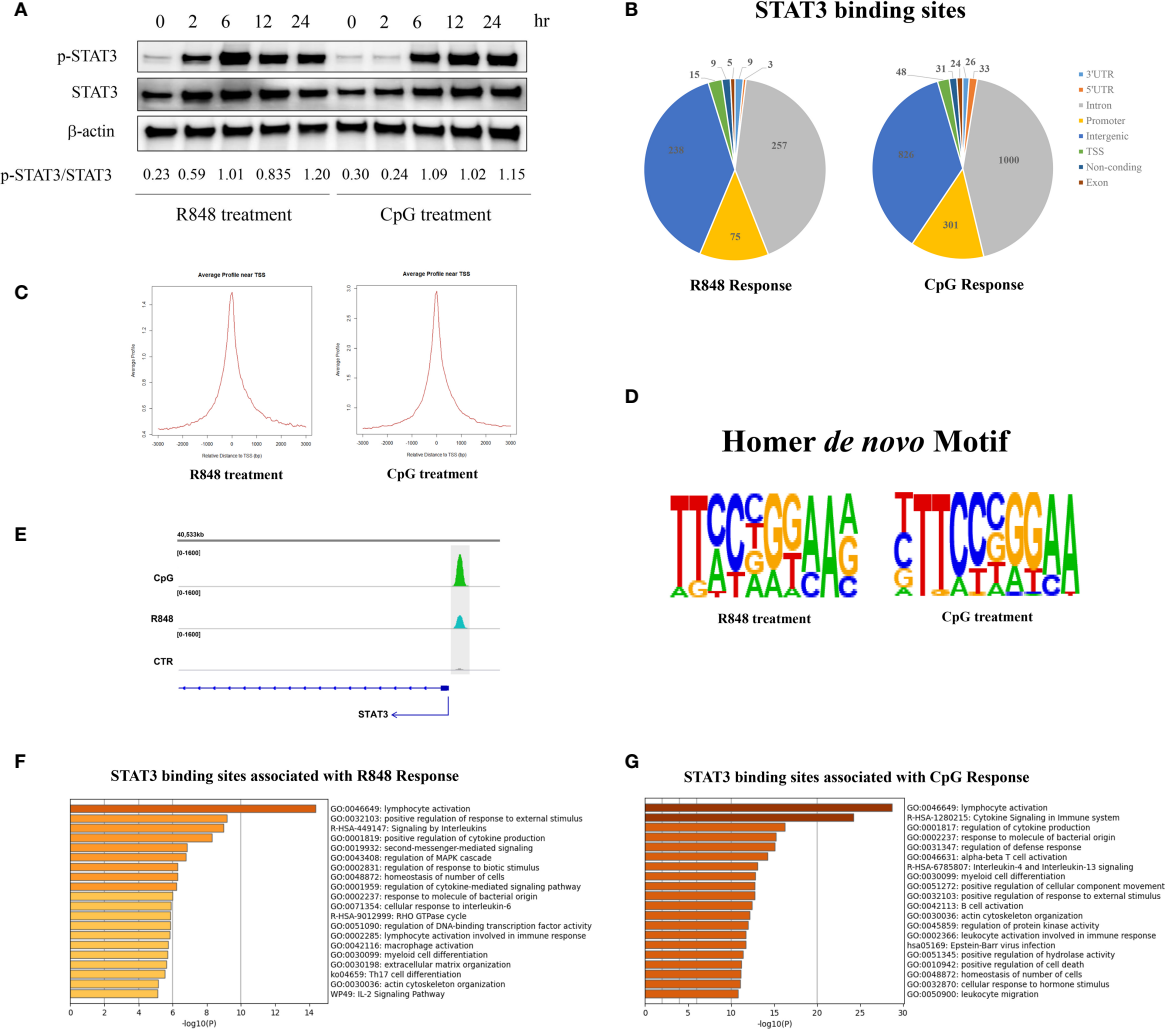
Properties of STAT3-binding sites genome-wide. **(A)** Western blot showing phosphorylation of STAT3 in response to TLR7 and TLR9 stimulation. Human peripheral blood B cells were treated with TLR7 and TLR9 agonists (R848 and CpG, respectively) for different timepoints (0, 2, 6, 12, and 24 h), then western blot was used to detect the phosphorylation of STAT3 (pSTAT3, Y705-STAT3). **(B)** ChIP-seq was performed on untreated, R848-treated and CpG-treated purified B cells from one healthy donor. The genomic distribution (%) of the identified STAT3-binding sites after 24 h treatment of R848 and CpG in B cells. STAT3-binding sites were identified by using the MACS tool. **(C)** Profiles of STAT3 binding sites are sharply peaked near the transcriptional starting site (TSS) of genes. Left, R848 stimulated vs non-stimulated B cells; Right, CpG stimulated vs non-stimulated B cells. **(D)** *De novo* motifs for R848 response and CpG response were identified from the STAT3 ChIP-seq binding sites by using Homer *de novo* motif analysis. **(E)** STAT3 binds to its own promoter in both R848 and CpG activated human peripheral blood B cells. Functional annotation resulting from the analysis of STAT3-binding sites in R848-mediated **(F)** and CpG-mediated **(G)** response in B cells with Metascape software.

To characterize the role of STAT3 in TLRs induced response in B cell, human purified B cells were treated with R848 and CpG for different timepoint (0, 2, 6, 12, and 24 h), and western blot was used to detected the phosphorylation levels of STAT3 (**Figure 1A**). The results showed that TLRs induced phosphorylation of STAT3 in human B cell, and the STAT3 phosphorylation induced by R848 was faster than that induced by CpG. In the present study, we are more interested to figure out the STAT3-regulated genes and its downstream signaling, which might be helpful for finding novel therapy to rescue or compensate the defective TLRs response in B cells of patients with AD-HIES. To visit this goal, we chose a long-term effect (24 h) mediated by STAT3 in TLRs-stimulated B cells in this study, which might be more acceptable for describing and reflecting the characteristics affected by STAT3.

After stimulation with CpG for 24h, ChIP-western blot was performed in human purified B cells with the anti-STAT3 antibody (IP), compared to the unfractionated crosslinked DNA–protein complexes (input) and unbound fraction (Elution) (**Supplementary Figure 1**). These results suggested that STAT3 is required for TLRs induced response in human peripheral blood B cell.

### STAT3-Binding Sites Are Specifically Associated With Immune Functions After TLRs Activation in B Cells

To characterize the regions bound by STAT3 after TLRs activation in B cells, STAT3 chromatin immunoprecipitation sequencing (ChIP-seq) studies, both in TLRs agonists-treated and control (CTR) cells, were carried out to identify the direct targets of STAT3. Model-based analysis for ChIP-seq (MACS) was used to identify peaks, and the analysis was performed on one treatment sample against a sample from a control experiment. A total of 611 and 2,289 differential STAT3-binding sites were identified in the R848 treated B cells and CpG treated B cells, respectively, indicating that STAT3 may directly regulate more genes upon CpG stimulation than R848 stimulation in human B cells after 24 h treatment. Among the binding sites, 495 (81.01%) and 1,826 (79.77%) were located in introns or intergenic regions, while only 90 (14.73%) and 349 (15.25%) were located in resided in transcription start sites (TSS) and promoter regions under R848 and CpG stimulation, respectively, indicating that STAT3 may regulate genes mostly through mechanisms that do not involve direct binding to promoters, but to distal regulatory elements (**Figure 1B**). Further analysis revealed that 40 and 42% of the STAT3 binding sites were localized within 10 kb up- or downstream of TSS in R848- and CpG-stimulated B cells, respectively (**Figure 1C**). Besides, *de novo* motif analysis using HOMER on all STAT3 binding peaks further revealed that a high conserved STAT3 homodimer motif (TTCCnGGAA) was top ranking in both R848-treated and CpG-treated B cells (p = 10^−134^ and 10^−386^, respectively) (**Figure 1D**). Notably, in line with the previous studies which showed STAT3 could bind to its own promoter (21, 22), we identified clear peaks at the promoter of the *STAT3* gene itself in both R848- and CpG-treated cells, but not in CTR cells in this study, indicating STAT3 is able to regulate B cell immune response upon R848 and CpG treatment through a positive regulatory loop (**Figure 1E**). This was consistent with our previous result (**Figure 1A**), which showed expression of STAT3 was increased after TLRs stimulation.

In the R848-stimulated B cells, 611 differential STAT3-binding sites were associated with 555 genes, and functional annotation of these 555 genes revealed a significant enrichment of terms associated with immune functions (**Figure 1F**). Similarly, in the CpG-stimulated B cells, 2,289 differential STAT3-binding sites were associated with 1,707 genes, and functional annotation also revealed a significant enrichment of terms associated with immune functions (**Figure 1G**). However, we have noticed some differences between the R848- and CpG-stimulated groups in this study. Firstly, the number of STAT3 directly targeted genes was significantly different between the two groups, which led to the number of genes in the given ontology term was remarkably different between the groups, and affected the P-values of the enriched terms. Besides, although majority of the enriched terms were same between groups, their order remarkably varied, which indicated that the activation of TLR7 and TLR9 had a quite different response in B cells *via* STAT3. Notably, some enriched terms were unique for R848- or CpG-stimulated group. For instance, Th17 cell differentiation pathway (ko04659) was enriched in R848-stimulated, but not in CpG-stimulated B cells, which was consistent with the previous report that strong TLR7 activation in pDCs and/or B cells may be required for T cell differentiation into Th17 (23).

### Comparison of STAT3 Directly Regulated Genes by TLR7 and TLR9 Agonists

**Figure 2:**
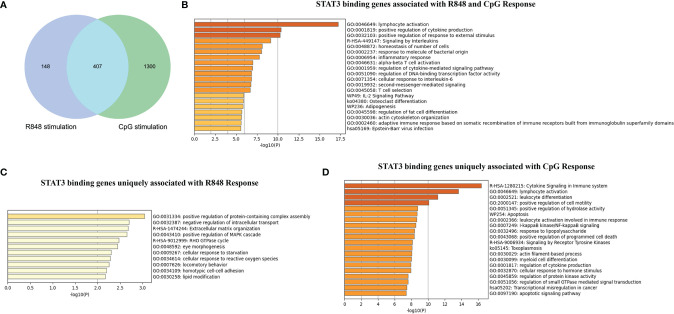
Comparison of STAT3 binding genes induced by TLR7 and TLR9 agonists stimulation in human B cells. **(A)** Venn diagram showing the overlap of STAT3 binding genes induced by R848 and CpG stimulation. Metascape software was use to perform functional annotation of overlapped STAT3- binding genes induced by R848 and CpG stimulation **(B)**, STAT3-binding genes uniquely induced by R848 stimulation **(C)**, and STAT3-binding genes uniquely induced by CpG stimulation **(D)**.

To further interpret the functional properties of the STAT3-binding sites genome-wide, we compared the STAT3 directly targeted genes in R848-stimulated and CpG-stimulated B cells. As shown in **Figure 2A**, 407 STAT3 directly targeted genes were identified as overlapping genes upon these two different TLRs agonists stimulations, and 148 genes were unique for R848 stimulation and 1,300 genes were unique for CpG stimulation. Further functional annotation of these 407 overlapping genes showed a significant enrichment of terms associated with immune response, such as “lymphocyte activation”, “positive regulation of cytokine production” and “positive regulation of response to external stimulus”, which indicated STAT3 played a vital role in immune response of B cells to various external TLR stimuli (**Figure 2B**). Interesting, STAT3 binding genes uniquely associated with R848 response and CpG response were enriched in substantially different ontology term, indicating that STAT3 also had a quite different role in response to R848 and CpG treatment in B cells (**Figures 2C, D**). The unique STAT3 binding genes for these two TLR signals in each of these pathways were listed in **Supplementary Tables 1** **,** **2**. Interesting, it was noted that STAT3 uniquely bind to more genes upon CpG stimulation, some of which have been well demonstrated to be vital for TLRs induced B cell activation, such as MyD88, CD40, CD86, AICDA, and so on (**Supplementary Figure 2**). This result could help to explain the defective TLR9 response in B cells from AD-HIES patients, namely, proliferation, activation, and immunoglobulin secretion. However, the unique STAT3 binding genes after R848 stimulation is significantly less than that after CpG stimulation, and they were enriched in some ontology terms which were less reported to be involved in TLRs response in B cells, such as regulation of small GTPase mediated signal transduction (NOTCH2, KALRN, FAM13B, ARHGAP17, PLEKHG4B, GPR199P, LEMD3, BAIAP2L2, and FMNL2), positive regulation of ERK1 and ERK2 cascade (FGB, NOTCH2, RAP1B, GPNMB, MTURN, MAPK8IP2, LRRK2, KLF10, and MDFIC) and so on. These results suggested STAT3 might be more involved in TLR9-stimulated than TLR7-stimulated response in B cells, at least in the 24 h timepoint.

Overall, our results suggest that STAT3-binding genes regulate key immune cell functions upon TLRs stimulation in B cells, and STAT3 might play an important, but not quite the same function in response to different TLRs stimulation.

### Transcriptome Sequencing Unveils Transcriptional Regulatory Networks in TLRs Induced Response in B Cells

**Figure 3:**
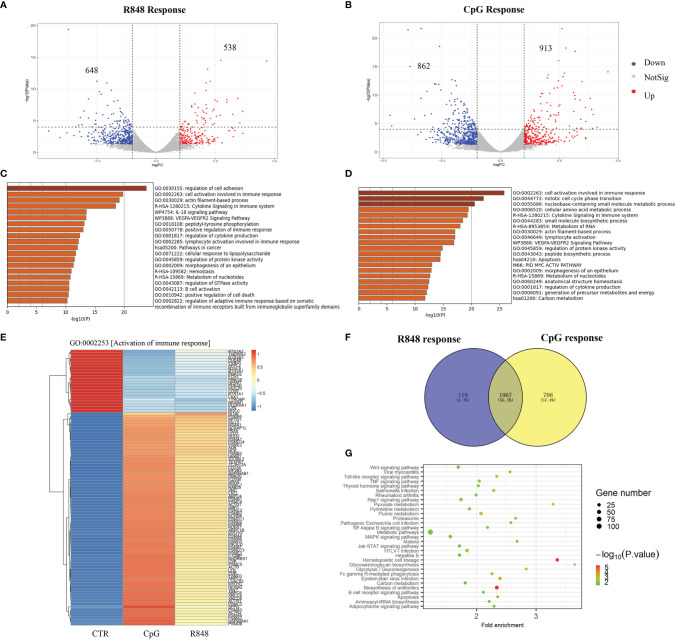
Transcriptome sequencing in TLRs induced response in B cells. RNA-seq with triple biological repeats was performed on untreated, R848-treated and CpG-treated purified B cells from three healthy donors. Volcano plot showing 1,186 differentially expressed genes (DEGs) in B cells upon R848 stimulation **(A)** and 1775 DGEs upon CpG stimulation **(B)**. Metascape software was use to perform functional annotation of DEGs induced by R848 stimulation **(C)** and CpG stimulation **(D)**. **(E)** Heatmap showing the DEGs in the biological progress gene ontology (GO) term “Activation of immune response”. **(F)** Venn diagram showing the overlap of DEGs induced by R848and CpG stimulation. **(G)** Visualization and Integrated Discovery (DAVID) was used to analyze Kyoto Encyclopedia of Gene and Genome (KEGG) pathway of DEGs overlapped in response to R848 and CpG stimulation.

Gene expression changes in response to TLRs agonists in human B cells were detected by comparing high-throughput RNA sequencing (RNA-seq) results on TLRs agonists-treated and untreated B cells. As shown in **Figure 3A**, a total of 1,186 genes were significantly changed after R848 stimulation, of which 538 genes were upregulated, and 648 genes were downregulated. More gene expression changes (1,775 genes) were observed in B cells after CpG stimulation, of which 913 genes were upregulated, and 862 genes were downregulated (**Figure 3B**). Interesting, these results were accordant with our ChIP-seq data, which showed more regions bound by STAT3 after CpG stimulation than R848 stimulation in B cells, thus indicated a noteworthy different transcriptome of B cells in response to TLRs activation. Functional annotation of these differentially expressed genes revealed that the enriched terms had remarkable difference between groups (R848 vs CTR and CpG vs CTR), demonstrating the different responses of B cells after different TLRs activation (**Figures 3C, D**). However, it could be noted that there were significant enrichments of terms associated with immune-related GO terms in both of the groups, and further analysis of the biological process GO term “activation of immune response” revealed similar gene expression changes in B cells after different TLRs activation (**Figure 3E**).

To explore the crosstalk between TLR7 and TLR9 signaling in B cells, we compared differentially expressed genes from R848-stimulated and CpG-stimulated B cells. As shown in **Figure 3F**, 1,067 differentially expressed genes were overlapped between groups, 119 genes were unique for R848-stimulated B cells, and 708 genes were unique for CpG-stimulated B cells. Expectedly, further Kyoto Encyclopedia of Genes and Genomes (KEGG) pathway enrichment analysis revealed an enrichment of some signaling pathway which has been demonstrated to be important for TLRs-induced B cell activation, such as “NF-kappa B signaling pathway”, “MAPK signaling pathway”, “Jak-STAT signaling pathway”, and “Fc gamma R-mediated phagocytosis” (**Figure 3G**). Notably, it was shown that an enrichment of some signaling pathways which have not been reported to be involved in TLRs-induced B-cell activation, namely, “Wnt signaling pathway”, “proteasome” and some metabolism pathway. Further studies are still needed to determine the contribution of these unreported pathways to the TLRs activation in B cells.

### Comparison of Transcriptional Regulatory Networks Induced by TLR7 and TLR9 Agonists in B Cells

**Figure 4:**
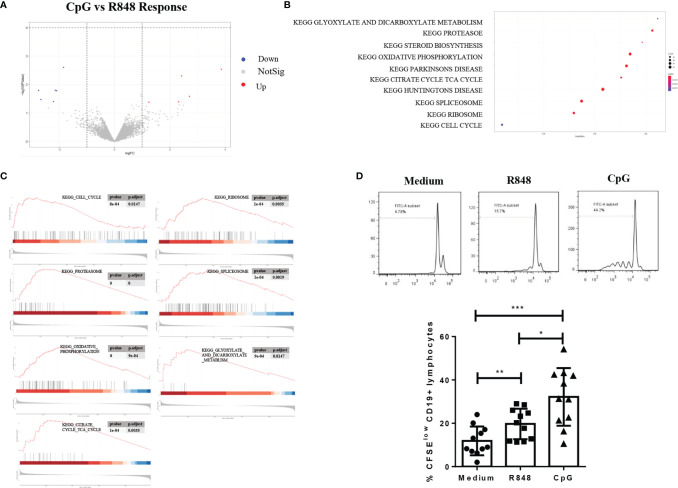
Comparison of transcriptional regulatory networks after TLRs activation in B cells. Volcano plot showing 13 differentially expressed genes (DEGs) in B cells upon CpG versus R848 stimulation **(A)**. GSEA analysis was performed on the CpG- versus R848-stimulated B cells, and the top 10 gene sets are shown **(B)**. GSEA analysis on the KEGG pathway of “Cell cycle”, “Ribosome”, “TCA cycle”, “Spliceosome”, “Oxidative phosphorylation”, “Glyoxylate and dicarboxylate metabolism and proteasome” and “TCA cycle” are shown **(C)**. The proliferation of CD19^+^ B cells after R848 and CpG stimulation was determined by flow cytometry using CFSE. The results were expressed as the mean percentage of proliferative CD19^+^ B cells ± SD, and each symbol represents an individual subject (n = 11) **(D)**. Statistical analysis was performed by one-way ANOVA, and *P < 0.05, **P < 0.01, and ***P < 0.001.

To investigate the difference of transcriptional regulatory networks induced by TLR7 and TLR9 agonists in B cells, we compared the differently expressed genes in R848-stimulated and CpG-stimulated B cells. As shown in **Figure 4A** and **Supplementary Table 3**, although only few genes were significantly differently expressed in the two groups, the further GSEA analysis showed that 29 gene sets were enriched in CpG-stimulated B cells, while 4 ontology gene sets were significantly enriched in R848-stimulated B cells. The top 10 gene sets are shown in **Figure 4B**. The highly populated pathways included those involved in protein synthesis (ribosome, spliceosome and proteasome), ATP generation (oxidative phosphorylation, TCA cycle, glyoxylate and dicarboxylate metabolism) and cell cycle (**Figure 4C**), which indicated CpG-stimulated B cell was more responsive to R848-stimulated B cell. Consistently, further study showed that the proliferation of B cell induced by CpG was significantly higher than that induced by R848 (**Figure 4D**).

### Identification of Immediate Target Genes of STAT3 After TLRs Activation in B Cells

**Figure 5:**
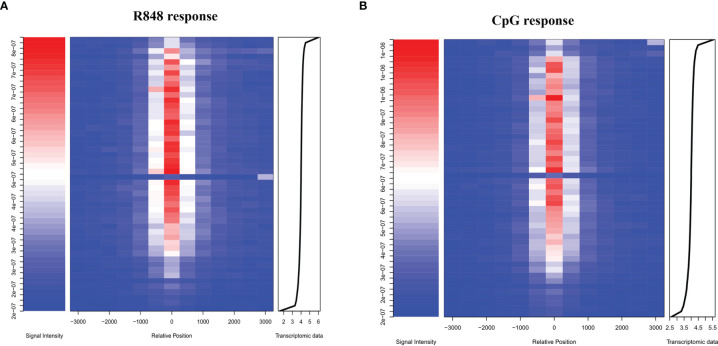
Identification of immediate target genes of STAT3 after TLRs activation in B cells. Heatmap showing the mean STAT3 ChIP-seq signal near TSS in function of transcriptomic data. The distribution of STAT3-binding sites after TLR7 **(A)** and TLR9 **(B)** activation in B cells (middle) and the peak signal densities (left) are shown. Transcriptomic data of B cell after TLRs stimulation were aligned from highest to lowest fold change of gene expression, and the x-axis coordinate of the transcriptomic data was log (FC ∗ 10,000) (right).

Identification of direct STAT3 target genes was carried out by combining ChIP-seq results with the list of differentially expressed genes after TLR activation in B cells. Heatmap in **Figures 5A, B** showed the mean STAT3 ChIP-seq signal near TSS in function of transcriptomic data. Remarkably, these results indicated us that STAT3 played a vital role, which not only directly upregulated, but also directly downregulated many genes which were involved in TLRs response in B cells. However, we observed an evident difference in the integration results (STAT3 ChIP-seq dada and RNA-seq data) from R848-stimulated and CpG-stimulated B cells, suggesting that the role STAT3 in B cell response to different TLRs agonists was not exactly the same.

### Comparison of Immediate Target Genes of STAT3 After TLRs Activation in B cells

**Figure 6:**
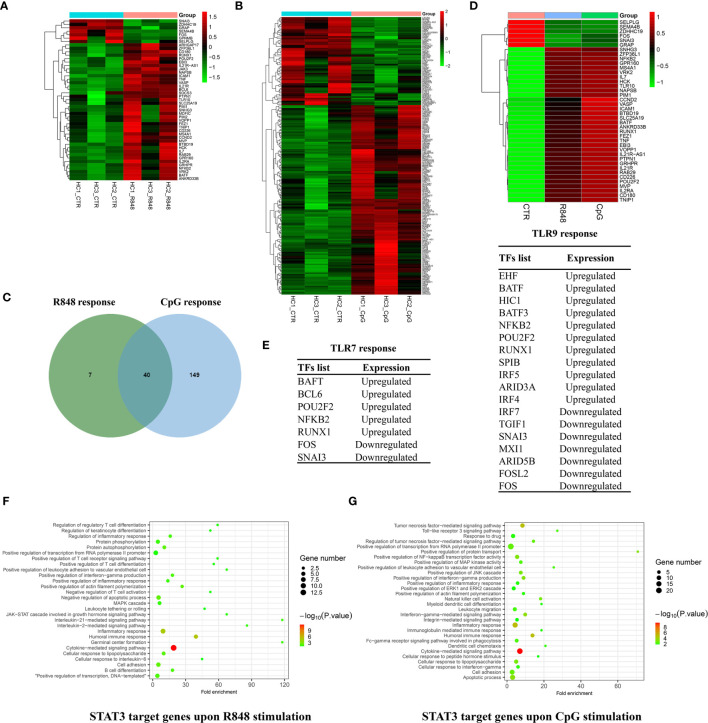
Comparison of immediate target genes of STAT3 after TLRs activation in B cells. Heatmap showing 47 STAT3 binding genes in response to R848 stimulation **(A)** and 189 STAT3 binding genes in response to CpG stimulation **(B)** within 20 kb of their TSS. **(C)** Venn diagram showing the overlap of STAT3 directly regulated genes induced by R848 and CpG stimulation. **(D)** Heatmap showing 40 overlapped STAT3 directly regulated genes in response to R848 and CpG stimulation. **(E)** Gene list showing STAT3 directly regulated TFs in response to R848 simulation and CpG simulation in B cells. Visualization and Integrated Discovery (DAVID) was used to analyze biological progress (GO) terms of immediate target genes of STAT3 after TLR7 **(F)** and TLR9 **(G)** activation in B cells.

From the list of differently expressed genes, we selected those with a STAT3-binding site within 20 kb of their TSS to end up with a list of 47 and 189 genes for R848 stimulation and CpG stimulation, respectively (**Figures 6A, B**). Forty genes were identified to be overlapped between the two groups, in which 6 genes were downregulated and 34 genes were upregulated after TLRs activation (**Figures 6C, D**). Only 7 genes (ARHGAP17, BCL6, GPNMB, JAK3, MDFIC, PIM2, SOCS3) were unique for TLR7 response, while 149 genes for TLR9 response, indicating that STAT3 might be more involved in TLR9 response in B cells.

To annotate the changes in the transcriptional factors (TFs) repertoire in response to TLRs activation, the set of TFs were defined according to the previous study (24). As shown in **Figure 6E**, we identified 7 TFs that were regulated on R848 stimulation and 18 TFs that were regulated on CpG stimulation, which were potentially directly regulated by STAT3. In a word, our results suggested STAT3 might regulated TLRs response in B cells through directly targeted some TFs.

The synergy and antagonism between TLR7 and TLR9 in B cells, raise a question that how they mediate signaling crosstalk, and what role STAT3 plays in this process. Functional annotations of STAT3 directly target genes responded to R848 and CpG stimulation are shown in **Figures 6F, G**. It was noted that an enrichment of terms, which were associated the biological process of immune response, in both of the two groups, such as “humoral immune response”, “inflammatory response”, “positive regulation of transcription from RNA polymerase II promoter”, “apoptotic process”, and so on. These terms and related genes, which are highly related to the B-cell response to TLRs stimulation, which provided informative clues to understand the defective TLRs response in B cells from AD-HIES patients with *STAT3* mutations (6, 7, 13). Besides, it is interesting to find the immediate target genes of STAT3 after CpG activation, but not R848 activation in B cells were significantly enriched in integrin-related biological progress, such as “integrin-mediated signal pathway”, “cell adhesion” and “cell migration”, indicating that STAT3 might be involved in integrin regulated TLR9, but not TLR7 response in B cells.

### STAT3 Regulated *IL-7* Expression in TLR-Stimulated B Cells by Directly Binding to its Promoter

**Figure 7:**
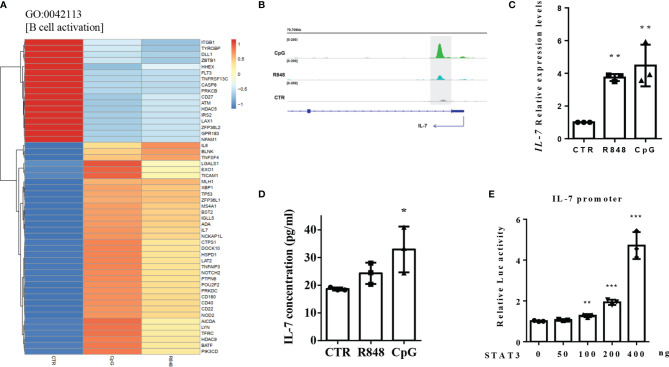
STAT3 regulated IL-7 expression by directly binding to its promoter. **(A)** Heatmap showing the TLRs-regulated genes in the biological progress (GO) term “B cell activation”. **(B)** STAT3 binds to *IL-7* gene promoter in TLR7/9 agonists activated human peripheral blood B cells. **(C)** RT-qPCR was used to detect *IL-7* expression in B cells in response to R848 and CpG stimulation. **(D)** Elisa assay was used to detect the concentrations of IL-7 in the supernatant of unstimulated (CTR), R848-stimulated and CpG-stimulated B cells. The data are presented as the mean ± standard deviation (SD) (n = 3). **(E)** Luciferase reporter assay was used to detect the effect of STAT3 on the luciferase activity of *IL-7* gene promoter. Three independent experiments were performed, and data are shown as mean ± SD. *P < 0.05, **P < 0.01, and ***P < 0.001.

Further analysis of the biological process GO term “B cell activation” showed several genes were differently expressed after both TLR7 and TLR9 activation (**Figure 7A**). Of these differently expressed genes, Interleukin 7 (*IL-7*) is particularly interesting because there is no evidence in the literature that *IL-7* can be produced by peripheral B cells. *IL-7*, which was involved in B-cell activation, was reported to play a certain role in early human B lymphopoiesis. In this study, clear peaks at the promoter of the *IL-7* gene were identified in both R848- and CpG-treated cells, but not in CTR cells (**Figure 7B**), indicating STAT3 might directly regulated its expression. As shown in **Figures 7C, D**, TLRs agonist stimulation could not only significantly increase the expression of *IL-7* genes in RNA levels, but also significantly increased the *IL-7* protein levels in B cells. Besides, STAT3 could upregulated *IL-7* gene promoter-driven luciferase activity (**Figure 7E**). These results indicated that STAT3 regulated *IL-7* expression by directly binding to its promoter.

## Discussion

The vital role of STAT3 in TLRs signaling in B cells has been well demonstrated, especially in AD-HIES patients with *STAT3* mutations whose TLRs response in B cells was shown to be defective (2, 6, 25). However, the molecular mechanism by which STAT3 influences downstream genes in TLRs signaling in B cells has not been identified on a genome-wide level. Figuring out the STAT3-regulated genes and its downstream signaling is very important for finding novel therapy to rescue or compensate the defective TLR response in B cells of patients with AD-HIES. Besides, STAT3 is not only involved in TLRs-mediated, but also BCR- and cytokine-mediated immune responses of B cells, which makes it more particularly interesting to identify STAT3 targets. It is therefore essential to examine the targets of STAT3 that may affect the TLRs response in human B cells. Herein, in this study, we identified the primary targets of STAT3 that are likely to play important roles in the TLRs-induced response in human B cells, and orchestrating the downstream process by influencing other TFs. STAT3 ChIP-seq revealed a mapping of 611 and 2,289 differential STAT3-binding sites in human B cells after TLR7 and TLR9 agonists stimulation, respectively. Besides, 1,186 and 1,775 differentially expressed genes were identified in human B cells after TLR7 and TLR9 activation, respectively. Among the 47 primary STAT3 target genes after TLR7 activation and 189 target genes after TLR9 activation in B cells, we identified 7 TFs and 18 TFs for TLR7 and TLR9 response. Finally, we showed that STAT3 might regulate TLR9, but not TLR7 response in B cells through directly regulating integrin signaling pathway, further affecting the antagonism between TLR7 and TLR9 signaling in B cells. These data suggest that STAT3 plays a vital, but not an exactly same role in different TLRs responses in B cells, which also provide informative clues to understand the defective TLR response in B cells from AD-HIES patients with *STAT3* mutations.

In line with the previous genome-wide level studies regarding STAT3-mediated transcription (21, 22), our results showed that approximately 80% STAT3 binding sites were located in introns or intergenic regions, suggesting that STAT3 may regulate gene expression mostly through distal regulatory elements. In fact, the presence of transcriptional activities in these non-coding regions has been well established in the previous studies (26, 27). However, up to now, little is known how STAT3 binding to non-coding regions regulates gene transcriptions, in which epigenetic regulation and shaping chromatin structures might be involved.

In this study, we focused on determining STAT3 target genes in B cells upon TLR7 and TLR9 stimulation. Mutations in *STAT3* gene has been reported to cause AD-HIES in the setting of a loss of functionality, but dysregulation of the immune system (such as immunodeficiency, malignancy, and autoimmunity) in the setting of a gain of functionality (11, 12, 28, 29). As natural models for determining the functions of STAT3 in TLRs signals in human B cells, AD-HIES patients were reported to have defective TLR9 response in B cells, namely, proliferation, differentiation, and immune globulin secretion, which provided very solid evidence on the importance of STAT3 in TLRs signals in human B cells (6, 7, 13). We further demonstrated that, not only TLR9, but also TLR7 response was defective in B cells from AD-HIES patients, further indicating the vital role of STAT3 in TLRs signals in human B cells (13). Notably, TLR7 and TLR9 activation were reported to have both synergistic and antagonistic effects in B-cell immune response, and a balance between TLR7 and TLR9 is pivotal in the development of B-cell autoreactivity. Our results identified 407 STAT3 directly target genes were overlapped in B cells upon TLR7 and TLR9 agonists stimulation, which were significantly enriched in the terms associated with immune response. Besides, 148 STAT3 target genes were identified to be unique for R848 stimulation and 1,300 genes for CpG stimulation. It was noted that STAT3 uniquely bind to more genes upon CpG stimulation, suggesting that STAT3 might be more involved in TLR9-stimulated than TLR7-stimulated response in B cells, at least in the 24 h timepoint. Notably, MyD88, which was reported to be an adaptor of TLRs signaling, was shown to be directly binded by STAT3 upon CpG stimulation. As it is well known that TLR9 induced STAT3 phosphorylation is through a MyD88-dependent mechanism, it could speculate that there is a positive feedback between MyD88 and STAT3 in B cells upon TLR9 stimulation. Taken together, the crosstalk and discrepancy mediated by STAT3 in TLR7 and TLR9 activation might contribute to their synergistic and antagonistic effects in B cells.

The RNA-seq data in this study showed that CD19^+^ B cells from human peripheral blood similarly respond to TLR7 and TLR9 agonists stimulation in regard to B-cell immune response, namely, B-cell activation, proliferation, migration, and cell survival through transcriptomic regulation, which was consistent with this previous study (30). This is not a surprise because both TLR7 and TLR9 agonists are nucleic acid, and B cells have a similar response to this kind of invasion. In fact, besides of JAK-STAT signal pathway, it was interesting for us to find an enrichment of some signaling pathways which have not been reported to be involved in TLRs-induced B-cell activation, namely, “Wnt signaling pathway”, “proteasome” and some metabolism pathways. These results highlighted that TLRs induced B-cell response was a quite complicated course, which might need a cooperation of transcriptomic, proteomic, and metabolomic regulation. However, it was noted that there was a significant difference in the enriched GO terms between R848-stimulated B cells and CpG-stimulated B cells, which could explain the different B-cell responses upon R848 and CpG stimulation. Moreover, the GESA analysis showed a significantly different enrichment for the ontology gene sets in R848-stimulated versus CpG-stimulated B cells, which further demonstrated the different B-cell responses upon R848 and CpG stimulation. Overall, the transcriptomic study of TLRs response in B cells provided a molecular framework for a better understanding of it.

Due to the vital role of STAT3 in multiple processes, namely, early development, cellular proliferation, survival and differentiation, it is necessary to identify the STAT3 targets for the further development of its intervention agents. More interesting, recent studies reported germline activating STAT3 led to autoimmune disease, while germline inactivating STAT3 mutations resulted in AD-HIES (31), emphasizing the critical role of STAT3 in the immune system. Integration of ChIP-seq and RNA-seq is a good strategy to identify STAT3 targets, which has been widely used in the previous studies (21, 22). Compared with TLR7 stimulation, we identified more STAT3 targets within 20 kb of their TSS in TLR9-stimulated B cells, which was consistent with our RNA-seq results and ChIP-seq results. Besides, in line with the previous studies, we identified 7 TFs that were regulated on R848 stimulation and 18 TFs that were regulated on CpG stimulation, suggesting that STAT3 could regulate TLRs response in B cells through regulating some TFs. Notably, some of the TFs, such as POU2F2 and RUNX1, were used reported to be able to bind to *STAT3* promoter, further regulating its expression. It indicated that there may be a feedback system between these TFs and gene transcription in TLRs response in B cells.

Given the antagonism between TLR7 and TLR9 signaling in B cells, it is interesting to figure out if STAT3 plays a role in this antagonism. Notably, in this study, we found that STAT3 might be involved in integrin regulated TLR9 response in B cells. αv integrin have long been known to be involved in clearance of apoptotic cells and other cellular debris which provide a rich source of autoantigens and nucleic acid TLR ligands (32). Previous reports have showed that integrin αvβ3 promoted the trafficking and maturation of TLR9-containing endosomes, and through this mechanism, reduced polyclonal activation of B cells through TLR9 (33). Therefore, we speculated that STAT3 might regulate TLR9, but not TLR7 response in B cells through directly regulating integrin signaling pathway, further affecting the antagonism between TLR7 and TLR9 signaling in B cells. Aberrant activation of STAT3 due to a gain of functionality might lead to an imbalance of TLR7 and TLR9 response through accelerating integrin signaling pathway, further affecting the development of autoimmunity.

Our studies still have some limitations. Firstly, we identified TLR-induced different gene expressions and STAT3 targeted genes in B cells after 24 h treatment with TLRs agonists in this study. Due to the original purpose of this study is to determine the molecular mechanism of defective TLRs response in B cells from AD-HIES with *STAT3* mutations, herein, we chose a long-term effect induced by STAT3 in TLRs-stimulated B cells, which might be more acceptable for describing and reflecting the characteristics affected by STAT3. However, a short time stimulation, or detecting TLR-induced different gene expressions and STAT3 targeted genes at different timepoints would be more informative. Secondly, although pathway analysis suggests that there are distinct responses to these TLR signals, these are not validated by further experimental approaches. All these works will be done in our further studies.

In summary, this is the first characterization of the genomic targets of STAT3 in responding to TLRs activation in B cells. Altered TLRs response and STAT3 activity in B cells are not only involved in immunodeficiency, but also autoimmune diseases. Main results obtained included identification of the STAT3-binding sites *in vivo*, the transcriptional changes, and the prediction of STAT3 targets associated with TLRs response in B cells. Our study provides insights into the molecular mechanism of human TLRs response in B cells and how it can be regulated, which provides a better understanding and modulating TLR-mediated pathogenic immune responses in B cells.

## Data Availability Statement

The datasets presented in this study can be found in online repositories. The names of the repository/repositories and accession number(s) can be found below: https://ngdc.cncb.ac.cn/gsa-human/, HRA001909.

## Ethics Statement

The studies involving human participants were reviewed and approved by the Shanghai Children’s Medical Center, School of Medicine, Shanghai Jiao Tong University. Written informed consent to participate in this study was provided by the legal guardian/next of kin of the participants.

## Author Contributions

T-XC, JW, and Y-YJ contributed to the designing and conducting the research, analyzing data, and writing the manuscript. R-LG, FY, and X-YS contributed parts of the experiments. All authors listed have made a substantial, direct, and intellectual contribution to the work and approved it for publication.

## Funding

This research was supported by grants from the National Natural Science Foundation of China (81701626, 81871303, 81571605, and 81273314).

## Conflict of Interest

The authors declare that the research was conducted in the absence of any commercial or financial relationships that could be construed as a potential conflict of interest.

## Publisher’s Note

All claims expressed in this article are solely those of the authors and do not necessarily represent those of their affiliated organizations, or those of the publisher, the editors and the reviewers. Any product that may be evaluated in this article, or claim that may be made by its manufacturer, is not guaranteed or endorsed by the publisher.

## Supplementary Material

Supplementary Figure 1 Western blot analysis of STAT3 chromatin immunoprecipitation (ChIP). Human peripheral blood B cell was treated with CpG for 24 h, then ChIP was performed using a specific antibody against STAT3, followed by immunoblot detection of STAT3. β-actin was used to detect the housekeeping protein in the input and unbound fractions.

Supplementary Figure 2 Reprehensive STAT3 binding sites unique for TLR7 and TLR9 activation. Results from the ChIP-seq were visualized in Integrative Genomics Viewer, and reprehensive STAT3 target genes unique for TLR7 **(A)** and TLR9 **(B)** activation were shown.
