# Supplementary figures and images for: Genome-Wide ChIP-seq and RNA-seq Analyses of STAT3 Target Genes in TLRs Activated Human Peripheral Blood B Cells
Source: Front Immunol. 2022 Mar 8;13:821457. doi: 10.3389/fimmu.2022.821457 (PMC8957201; doi:10.3389/fimmu.2022.821457)

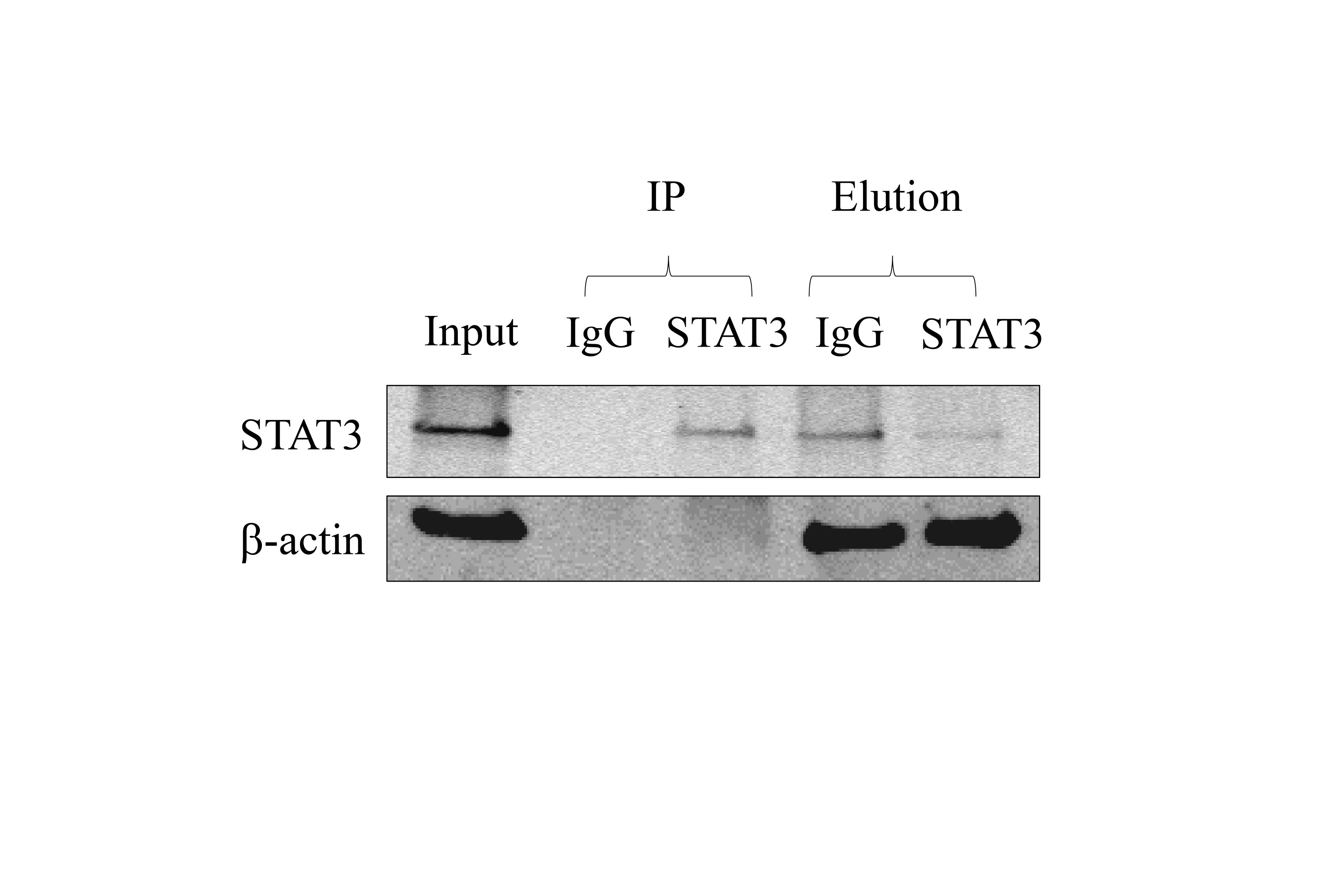

Supplement: Supplementary Figure 1 — Western blot analysis of STAT3 chromatin immunoprecipitation (ChIP). Human peripheral blood B cell was treated with CpG for 24 h, then ChIP was performed using a specific antibody against STAT3, followed by immunoblot detection of STAT3. β-actin was used to detect the housekeeping protein in the input and unbound fractions. [file Image_1.tif]

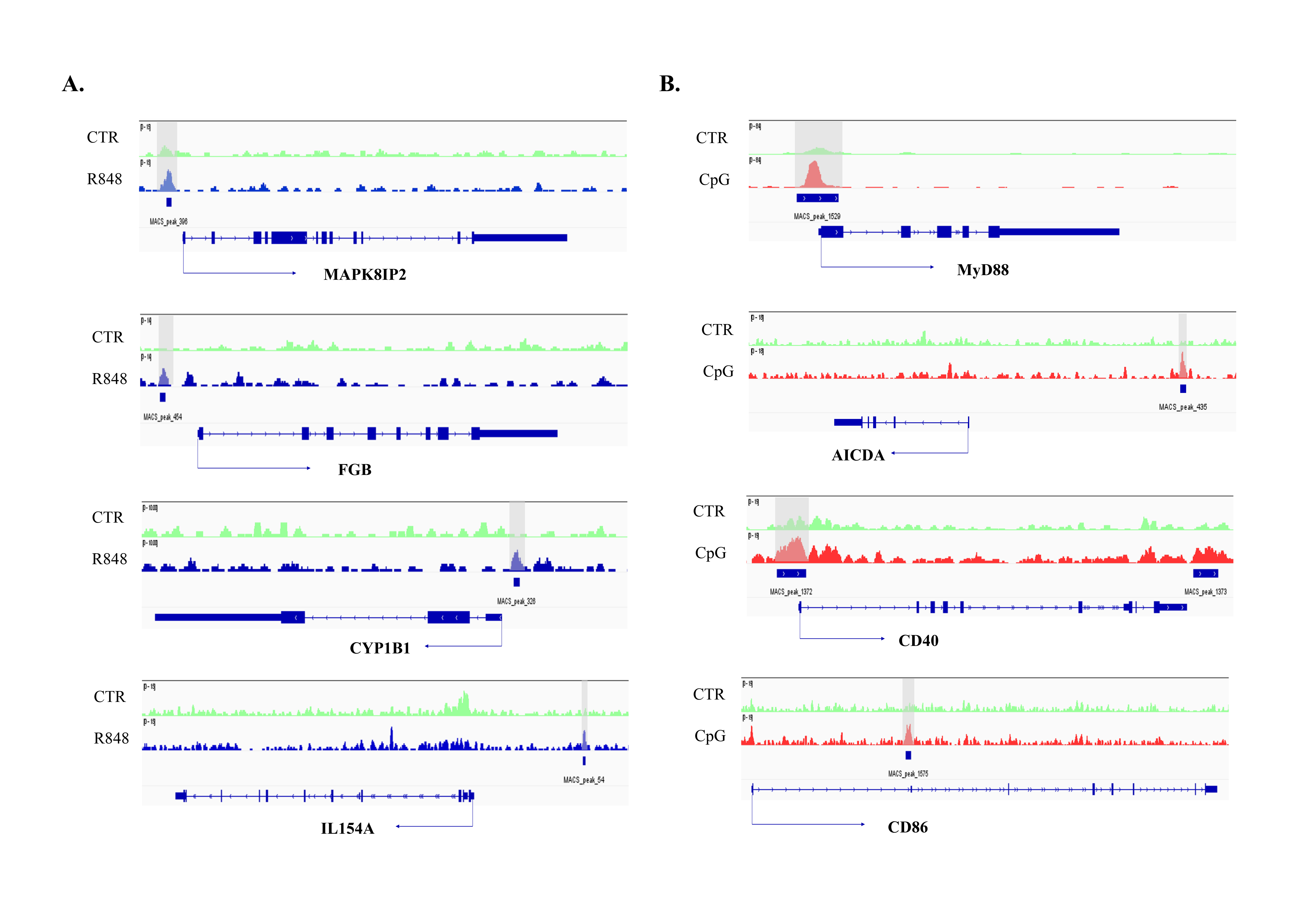

Supplement: Supplementary Figure 2 — Reprehensive STAT3 binding sites unique for TLR7 and TLR9 activation. Results from the ChIP-seq were visualized in Integrative Genomics Viewer, and reprehensive STAT3 target genes unique for TLR7 (A) and TLR9 (B) activation were shown. [file Image_2.tif]
