# Supplementary material for: Genome-Wide ChIP-seq and RNA-seq Analyses of STAT3 Target Genes in TLRs Activated Human Peripheral Blood B Cells
Source: Front Immunol. 2022 Mar 8;13:821457. doi: 10.3389/fimmu.2022.821457 (PMC8957201; doi:10.3389/fimmu.2022.821457)
Supplement: Supplementary file 3 [file Table_1.docx]

**Supplementary Table 1.** List of pathways obtained by gene set enrichment analysis (GSEA) of the Kyoto Encyclopedia of Genes and Genomes (KEGG) database

| **Name** | **Size** | **NES** | **p.adjust** |
| --- | --- | --- | --- |
| KEGG_PROTEASOME | 40 | 2.306737 | 1.37E-05 |
| KEGG_HUNTINGTONS_DISEASE | 133 | 1.962799 | 1.78E-04 |
| KEGG_OXIDATIVE_PHOSPHORYLATION | 88 | 1.969116 | 8.82E-04 |
| KEGG_PARKINSONS_DISEASE | 87 | 1.967082 | 9.50E-04 |
| KEGG_SPLICEOSOME | 124 | 1.811054 | 1.86E-03 |
| KEGG_RIBOSOME | 83 | 1.833525 | 3.49E-03 |
| KEGG_CITRATE_CYCLE_TCA_CYCLE | 27 | 2.050752 | 3.49E-03 |
| KEGG_STEROID_BIOSYNTHESIS | 14 | 2.018675 | 8.26E-03 |
| KEGG_CELL_CYCLE | 114 | 1.646953 | 1.47E-02 |
| KEGG_GLYOXYLATE_AND_DICARBOXYLATE_METABOLISM | 13 | 1.964271 | 1.47E-02 |
| KEGG_ALZHEIMERS_DISEASE | 124 | 1.623175 | 1.47E-02 |
| KEGG_VALINE_LEUCINE_AND_ISOLEUCINE_DEGRADATION | 40 | 1.792528 | 1.79E-02 |
| KEGG_PORPHYRIN_AND_CHLOROPHYLL_METABOLISM | 20 | 1.926121 | 1.95E-02 |
| KEGG_PYRIMIDINE_METABOLISM | 87 | 1.61661 | 2.74E-02 |
| KEGG_TYPE_I_DIABETES_MELLITUS | 32 | 1.763836 | 2.81E-02 |
| KEGG_CYTOKINE_CYTOKINE_RECEPTOR_INTERACTION | 133 | 1.530631 | 3.12E-02 |
| KEGG_BIOSYNTHESIS_OF_UNSATURATED_FATTY_ACIDS | 17 | 1.792033 | 3.17E-02 |
| KEGG_RNA_DEGRADATION | 53 | 1.639879 | 3.17E-02 |
| KEGG_AMINOACYL_TRNA_BIOSYNTHESIS | 22 | 1.824656 | 3.42E-02 |
| KEGG_PEROXISOME | 65 | 1.62455 | 3.42E-02 |
| KEGG_JAK_STAT_SIGNALING_PATHWAY | 92 | 1.57571 | 3.42E-02 |
| KEGG_TERPENOID_BACKBONE_BIOSYNTHESIS | 14 | 1.828459 | 3.42E-02 |
| KEGG_AMYOTROPHIC_LATERAL_SCLEROSIS_ALS | 41 | 1.675797 | 3.42E-02 |
| KEGG_PATHWAYS_IN_CANCER | 231 | -1.402694 | 3.42E-02 |
| KEGG_ONE_CARBON_POOL_BY_FOLATE | 15 | 1.829668 | 3.42E-02 |
| KEGG_BASAL_CELL_CARCINOMA | 32 | -1.638585 | 4.10E-02 |
| KEGG_ASTHMA | 17 | 1.7145 | 4.16E-02 |
| KEGG_NEUROACTIVE_LIGAND_RECEPTOR_INTERACTION | 65 | -1.59318 | 4.21E-02 |
| KEGG_RNA_POLYMERASE | 27 | 1.735568 | 4.21E-02 |
| KEGG_HOMOLOGOUS_RECOMBINATION | 25 | 1.728141 | 4.65E-02 |
| KEGG_PROTEIN_EXPORT | 22 | 1.73515 | 4.81E-02 |
| KEGG_ECM_RECEPTOR_INTERACTION | 39 | -1.597952 | 4.81E-02 |
| KEGG_O_GLYCAN_BIOSYNTHESIS | 15 | 1.767513 | 4.84E-02 |
